# Medical and Surgical Reporter, Philadelphia

**Published:** 1877-10-20

**Authors:** 


MEDICAL AND SURGICAL REPORTER," PHILADELPHIA.—This is among the very best of our large list of nearly fifty exchanges. See their Fall announcement in present issue.

